# Effects of different concentrations of biochar amendments and Pb toxicity on rhizosphere soil characteristics and bacterial community of red clover (*Trifolium pretense L*.)

**DOI:** 10.3389/fpls.2023.1112002

**Published:** 2023-03-28

**Authors:** Lingdong Meng, Yuchen Wu, Meiqi Mu, Zicheng Wang, Zirui Chen, Lina Wang, Zewang Ma, Guowen Cui, Xiujie Yin

**Affiliations:** College of Animal Science and Technology, Northeast Agricultural University, Harbin, China

**Keywords:** lead stress, corn straw biochar, 16S rDNA amplicon sequencing, bacterial, red clover, soil enzyme activity

## Abstract

Amending soil with biochar can reduce the toxic effects of heavy metals (HM) on plants and the soil. However, the effects of different concentrations of biochar on the properties and microbial activities in lead (Pb)-contaminated soils are unclear. In this study, two Pb concentrations were set (low, 1000 mg/kg; high, 5000 mg/kg), and five corn straw biochar (CSB) concentrations (0, 2.5, 5, 10 and 15%) were used to determine the response of the growth and rhizosphere of red clover (*Trifolium pretense* L.) (in terms of soil properties and bacteria) to CSB and Pb application. The results showed that 5% CSB better alleviated the toxicity of Pb on the shoot length of red clover, the biomass increased by 74.55 and 197.76% respectively and reduced the enrichment factor (BCF) and transport factor (TF) of red clover. Pb toxicity reduced soil nutrients, catalase (CAT), acid phosphatase (ACP) and urease activity, while the addition of CSB increased soil pH, soil organic matter (SOM) content and soil enzyme activity. 16S rDNA amplicon sequencing analysis showed that Pb toxicity reduced the diversity of rhizosphere bacteria in red clover and reduced the relative abundance of plant growth-promoting rhizobacteria such as *Gemmatimonas*, *Devosia* and *Bryobacter*. Spearman correlation analysis showed that the addition of alkaline CSB restored the relative abundance of rhizobacteria positively correlated with pH, such as *Chitinophaga*, *Sphingomonas*, *Devosia* and *Pseudomonas*, and thus restored the rhizosphere soil environment. This study demonstrates that 5% CSB can better alleviate the toxicity of Pb to red clover and soil. We also provide a theoretical basis for the subsequent use of beneficial bacteria to regulate the repair efficiency of red clover.

## 1 Introduction

Soil accumulation of heavy metals (HM) from natural and human sources has become a major problem in terrestrial ecosystems (Mourad et al., 2021). The main human sources of these HM are metal mining activities, industrial activities, automobile exhaust emissions and wastewater irrigation. HM not only poison soils and water resources but also hinder plant productivity. HM can also accumulate in the food chain, ultimately threatening human health (Azadi and Raiesi, 2021). Lead (Pb) is one of the most common HM pollutants in the environment. The Pb concentration is approximately 50 mg/kg in the soil in nature (Bamagoos et al., 2022). However, due to the use of paint, Pb-containing gasoline and other Pb-containing products and the lack of effective treatment methods, the Pb concentration in the environment is much higher than the recommended safe concentration (Salam et al., 2016). In addition, Pb is nonbiodegradable and can persist in the soil environment for a long time (Kumar et al., 2020). Studies have shown that children have a stronger absorption capacity for Pb than adults, and Pb exposure can cause cognitive, hearing and cardiovascular problems (Fewtrell et al., 2004). Physical and chemical remediation methods for removing Pb from contaminated soils can reduce Pb toxicity and mitigate the risk of Pb exposure (Xinde et al., 2011). However, widespread application of these soil remediation techniques is limited due to high costs and the destruction of the soil structure (Muthusaravanan et al., 2018). Therefore, identifying economic, efficient and environmentally friendly sustainable soil remediation techniques is urgently needed.

Biochar is a carbon-rich porous material produced *via* pyrolysis of biomass under anaerobic conditions (Uchimiya et al., 2010). As a soil conditioner, biochar can cause changes in soil physical and chemical properties and has been widely used in the remediation of HM-contaminated soil (Omidi et al., 2019; Yang et al., 2022). Studies have shown that, owing to its porous structure, biochar can adsorb and fix pollutants in the soil, reduce the bioavailability of HM (Zhao et al., 2021), and reduce both the migration of HM in the soil and the incorporation of HM from the soil into plants, thereby preventing HM from threatening human health *via* the food chain. It has been reported that biochar application has remediation effects on chromium, arsenic, Pb and cadmium. In addition, biochar can improve soil properties and increase both the soil pH and soil organic matter content (Qiu et al., 2021), thereby mitigating damage caused by HM and promoting plant growth; this has been shown for foxtail millet (Kang et al., 2022) and wheat (Yanru et al., 2022). There are many studies on the effects of the pyrolysis temperature of biochar on its ability to improve Pb-contaminated soils (Chi et al., 2017; Yang et al., 2021); however, the application amount of biochar may be the most important factor affecting its ability to remediate Pb. Jing et al. reported that wheat straw-derived biochar reduced the migration of Pb (Jing et al., 2020), and Cheng et al. showed that adding 5% tobacco straw-derived biochar reduced the available Pb concentration better than a low dose (Jianzhong et al., 2018). In China, the annual yield of corn straw is high, but the utilization rate is low. The imbalance between yield and utilization leads to a large amount of corn straw being discarded or burned, which wastes resources and pollutes the environment (Yang et al., 2019). Therefore, corn straw biochar (CSB) was used as a soil amendment to reduce Pb pollution.

Red clover (*Trifolium pretense* L.) is a legume perennial herb with high protein content, good palatability and low demand for nitrogen fertilizer (Chao et al., 2018). Previous studies have found that red clover has a strong tolerance to heavy metals and shows the ability to accumulate heavy metals (Jin et al., 2013). Therefore, it has received extensive attention in the remediation and detoxification of contaminated field (Cruz et al., 2021). Mo found that the photosynthetic capacity and antioxidant enzyme activity of red clover could maintain a certain level under heavy metal environment. In addition, the migration ability of roots to heavy metals was low (Mo et al., 2021). This means that it may have good potential for resistance to heavy metal toxicity.

Soil microorganisms may be the most sensitive soil organisms to subtle changes in the soil environment. Therefore, the toxic effect of Pb in the soil can reduce soil microbial biomass (Nahid and Fayez, 2021), inhibit microbial metabolism (Xu et al., 2021) and alter microbial community composition (Jianhua et al., 2021). Kamaruzzaman et al. found that Pb exposure enhanced the Pb resistance of microorganisms; for example, *Bacillus* and *Enterobacter* were found to have high tolerance to Pb stress (Kamaruzzaman et al., 2020). The application of biochar to cadmium (Cd)-contaminated soil increased the abundance of *Pseudomonas*, thereby reducing the absorption of Cd by plants. The porous structure and large surface area of biochar provide shelter for microorganisms (Chuchu et al., 2022). Soil pH plays an important role in the degradation of contaminants by influencing the activity of cells, the positive or negative surface charge of substances, HM solubility and the ionization state of functional groups. Microbes are vulnerable to the environmental pH balance. Under normal conditions, the optimum pH value for the reproduction and vivacity of most microorganisms is between 6.0 and 8.0 (Youyuan et al., 2021). At suitable pH values, *Bacillus* has a stronger ability to remove pollutants (Zhang et al., 2020). In addition, pH also affects the mobility of HM and biochar surface charges, thereby affecting the interaction between pollutants and biochar (Chuchu et al., 2022). Overall, the addition of biochar increased the relative abundance of C and N cycling-related microorganisms in the soil. However, the correlation between red clover growth, physiological changes and soil physical and chemical properties and the changes in rhizosphere soil bacteria under Pb toxicity are still unclear.

In this study, soil physicochemical properties and 16S rDNA amplicon sequencing were combined to elucidate the response mechanism of red clover rhizosphere soil to different concentrations of Pb and different application rates of CSB. The purposes of this study were (a) to investigate the effects of Pb toxicity and biochar amendments on soil physicochemical properties and the soil bacterial community; (b) to analyze the interactions between soil characteristics and the bacterial community; and (c) to determine the optimal biochar concentration for mitigating Pb toxicity red clover. Our results should provide new insights into the interactions between soil physicochemical properties and the soil bacterial community in response to different doses of biochar.

## 2 Materials and methods

### 2.1 Plant, soil, and corn straw biochar

In this study, the soil was collected from the experimental field of Northeast Agricultural University in Harbin, China (E 126°14′; N 45°05′), then quickly transported back to the laboratory, dried at 25°C, and keep the soil moisture at 60%. The surfaces of the selected red clover seeds were disinfected (Liu et al., 2021), washed three times with distilled water, and germinated in a dark environment at 25°C. Biochar was produced by pyrolysis of corn straw at 500°C using furnace equipment (LZ-LS-600*800-420, China). The initial soil pH, SOM, available phosphorus (AP) and available potassium (AK) were 6.13, 29.37 g/kg, 139.34 mg/kg and 59.06 mg/kg respectively. In addition, the CSB had a pH of 9.4 and Pb concentration of 8.12 mg/kg.

### 2.2 Experimental design

The Pb-containing soil was set to 1000 mg/kg (low lead) and 5000 mg/kg (high lead) with Pb(NO_3_)_2_, and 1 kg of soil was preserved in each pot. After two weeks of passivation, CSB was mixed with Pb-containing soil at five ratios, i.e., 0, 2.5, 5, 10 and 15%. Then, the plants were selected with consistent growth and planted 10 plants in a pot (planting environment: 16 h/8 h light/dark cycle, 25 ± 1°C) (Wang et al., 2022), 70% relative humidity (Zhang et al., 2021). In addition, impervious plates were placed under pots to absorb the leachate from the pots and return it to the soil every two days. The treatments were set as CK (0 mg/kg Pb + 0% CSB), LB0 (1000 mg/kg Pb + 0% CSB), LB2.5 (1000 mg/kg Pb + 2.5% CSB), LB5 (1000 mg/kg Pb + 5% CSB), LB10 (1000 mg/kg Pb + 10% CSB), LB15 (1000 mg/kg Pb + 15% CSB), HB0 (5000 mg/kg Pb + 0% CSB), HB2.5 (5000 mg/kg Pb + 2.5% CSB), and HB5 (5000 mg/kg Pb + 5% CSB). HB10 (5000 mg/kg Pb + 10% CSB) and HB15 (5000 mg/kg Pb + 15% CSB), with total of 11 treatments, and each treatment had 3 replicates. Red clover plants and soil samples were collected 45 days later. The loose soil was divided into bulk soil and collected the soil around the roots as rhizosphere soil for subsequent 16S rDNA amplicon sequencing.

### 2.3 Plant growth index and Pb content

After exposure to Pb stress for 45 days, the stem length of red clover was measured using a ruler with a precision of 1 mm. In the determination of Pb concentration, plant stems and roots were dried (105°C and 12h), and the soil was ground to a diameter of less than 0.02 mm. Then, 0.5 g of the sample was added to 37% HCl and 63% HNO_3_ (150°C) for digestion, diluted with HNO_3_, and finally analyzed by inductively coupled plasma−mass spectrometry (Agilent, 7800 ICP−MS, USA) (Helaoui et al., 2020).

### 2.4 The bioconcentration factor and translocation factor of Pb

Using the method reported by Meng (Meng et al., 2022), we calculated the bioaccumulation factor (BCF =Pb concentration in plant/Pb concentration in soil) and transport factor (TF =Pb concentration in shoot/Pb concentration in root).

### 2.5 Soil properties

According to the method of Liu et al., the pH and electrical conductance (EC) of the soil suspension (1:5, w/v) were measured by a pH meter and conductivity meter, respectively. SOM was determined by the dry combustion method (Liu et al., 2020). Available phosphorus (AP), available potassium (AK), ammonia nitrogen and nitrate nitrogen were measured by Lu et al. (Lu et al., 2000). Catalase, acid phosphatase, urease and sucrose (SC) levels in the rhizosphere soil of red clover were measured by using a kit purchased from Shanghai Enzyme-linked Biotechnology Co., Ltd. (www.mlbio.cn), and then measured the absorbance at 450 nm using a microplate reader (k6600-a, Beijing Kaiao Technology Development Co., Ltd).

### 2.6 Rhizosphere bacterial community analysis

Genomic DNA was extracted from rhizosphere soil by the CTAB/SDS method. Amplicons were amplified according to the manufacturer’s instructions as follows: initial denaturation at 98°C for 1 min, followed by denaturation at 98°C for 10 s (30 cycles), followed by annealing and extension at 50°C and 72°C for 30 s, respectively, and centrifugation at 72°C for 5 min. An equal volume of 1X loading buffer containing SYB green was mixed with PCR products and purified using a Qiagen Gel Extraction Kit (Qiagen, Germany). The TruSeq ^®^ DNA PCR-Free Sample Programming Kit (Illumina, USA) was used to generate sequencing libraries and add index codes as recommended by the manufacturer. Library quality assessment was performed on a Qubit @ 2.0 fluorometer (Thermo Scientific) and an Agilent Bioanalyzer 2100 system. Finally, the library was sequenced on the Illumina Nova Seq platform, resulting in 250 bp double-ended reads.

The raw tags were filtered with reference to the QIIME (V 1.9.1) tag quality control process (Caporaso et al., 2010). Compare the above tags with the SILVA database and use the UCHIME Algorithm (Brian et al., 2011) to detect the chimera sequence and remove it. Finally, obtain effective tags. The sequences were clustered into operational taxonomic units (OTUs) with 97% identity, and the OTU sequences were annotated. The species annotation analysis was performed using the Mothur method and SILVA138 (http://www.arb-silva.de/) SSUrRNA database (the threshold was set to 0.8–1) (Wang et al., 2007; Edgar, 2013).

### 2.7 Statistical analysis

Statistical calculations were carried out by Microsoft Office Excel 2016, Origin 2018, IBM SPSS version 26.0 software and R package. In addition to one-way ANOVA, the Wilcox test was used for analyzing bacterial richness and diversity (Bauer, 1972). The images were merged using Adobe Photoshop 2020. The correlation between the physicochemical properties of rhizosphere soil and rhizosphere bacterial community structure was analyzed by Spearman correlation analysis. SIMPER ranked OTUs according to the contribution rate of each treatment group to the difference score. The Silva database was used to identify 25 OTUs identified by SIMPER analysis of CK *vs*. HB0 and HB0 *vs*. HB5.

## 3 Results

### 3.1 Plant growth and accumulation and transport of Pb

**Figure 1:**
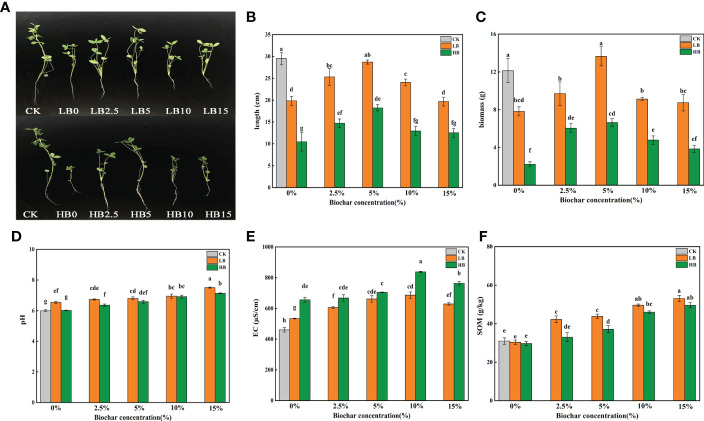
Effects of CSB at different concentrations on **(A, B)** shoot length of red clover, **(C)** biomass of red clover, **(D)** rhizosphere soil pH, **(E)** EC and **(F)** SOM under Pb toxicity. Different small letters indicate significant differences under different Pb concentration treatments and different biochar doses (P<0.05).

In this study, compared with CK, the stem length of red clover was significantly reduced by 32.78% and 64.48% at LB0 and HB0, respectively (P < 0.05). The application of CSB alleviated the toxic effect of Pb on the stem growth of red clover, and the effects were dependent on CSB concentration. Compared with LB0 and HB0, LB5, LB2.5, HB5 and HB2.5 significantly increased by 44.69, 27.61, 73.88 and 40.23%, respectively. However, the mitigation effects of 10% and 15% CSB on Pb toxicity decreased (**Figures 1A, B**). And the biomass change trend of red clover is similar to stem length, compared with LB0 and HB0, the biomass of LB5 and HB5 increased by 74.55 and 197.75%, respectively. (**Figure 1C**). Combining the data on Pb concentration in the soil and in red clover, we calculated the Pb enrichment factor (BCF) and transport coefficient (TF) of red clover (**Table S1**). High concentration of Pb caused a significant increase in the BCF of red clover plants, and the addition of CSB to the Pb-contaminated soil caused a downward trend in the BCF and TF. These results showed that Pb toxicity inhibited the growth of red clover, and the addition of CSB resulted in more fixing of Pb in the soil, alleviated Pb toxicity, reduced the Pb enrichment and transport capacity, and stimulated plant growth. Moreover, the remediation effect of 5% CSB was better than that of the other treatment groups.

### 3.2 Effects of Pb and CSB on soil physicochemical properties

At the same Pb concentration, the soil pH significantly increased with increasing CSB concentration (P < 0.05), and the highest soil pH values were recorded in LB15 (pH = 7.505) and HB15 (pH = 7.129) (**Figure 1D**). At the same CSB level, the pH of the low-Pb treatment was higher than that of the high-Pb treatment. When the Pb concentration in the soil was 1000 and 5000 mg/kg, the electrical conductivity (EC) was in the order of CK < LB0 < HB0. Compared with the levels of their respective control treatments, all CSB levels increased the soil EC regardless of Pb concentration, and the EC was highest in the two Pb concentration treatments when 10% CSB was added (**Figure 1E**).

Compared with the no-Pb stress treatment, the 1000 and 5000 mg/kg Pb concentration treatments had no significant effect on the SOM content. At the 1000 mg/kg Pb level, CSB application significantly increased the SOM content, and the highest SOM content was recorded in LB15 (53.12 g/kg). For the high Pb concentration treatments, the SOM contents of HB2.5, HB5, HB10 and HB15 were 10.86, 24.83, 54.67 and 66.72% higher than that of HB0, respectively (**Figure 1F**).

**Figure 2:**
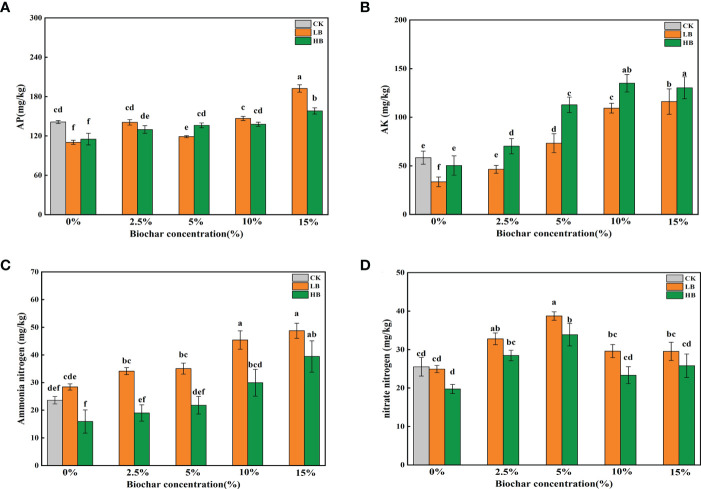
Effects of CSB at different concentrations on **(A)** available phosphorus, **(B)** available potassium, **(C)** ammonium nitrogen and **(D)** nitrate nitrogen under Pb toxicity. Different small letters indicate significant differences under different Pb concentration treatments and different biochar doses (*P*<0.05).

Pb treatment significantly reduced the soil AP content (P < 0.05) compared with that of the CK. Except for LB5, with the increase in CSB concentration, the AP content increased in the two Pb treatments; the AP content increased by 74.58 and 37.34% in LB15 and HB15, respectively, compared with LB0 and HB0, respectively (**Figure 2A**). The AK content under low Pb stress was significantly lower than that under the CK treatment, but no significant effect was found under high Pb stress. With the increase in CSB concentration, both Pb levels were in the following order: 0% < 0.5% < 5% < 10% < 15% (**Figure 2B**).

Under low Pb stress (1000 mg/kg), the soil ammonium-nitrogen content in the CSB-treated samples was higher than that under high Pb stress (5000 mg/kg). Compared with the CK treatment, low Pb stress promoted the soil ammonium-nitrogen content, but high Pb stress inhibited it, indicating that a high Pb concentration has a negative impact on the soil ammonium-nitrogen content; CSB application could alleviate this inhibition (**Figure 2C**). Adding CSB increased the soil nitrate-nitrogen content. When 5% CSB was applied, the soil nitrate-nitrogen content peaked, and the LB5 and HB5 nitrate-nitrogen contents were 29.53 and 23.33 mg/kg, respectively. An increasing CSB concentration decreased the nitrate-nitrogen content, indicating that the application of CSB can improve the soil nitrate-nitrogen level, but there was a limit, as excessive CSB concentrations result in too much nitrate-nitrogen inhibition (**Figure 2D**).

### 3.3 Effects of Pb and CSB on soil enzyme activity

**Figure 3:**
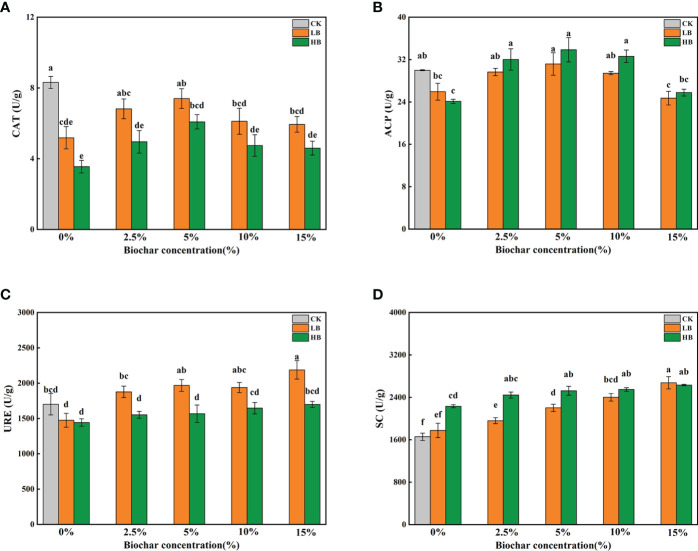
Effects of different concentrations of CSB on enzyme activities in Pb-contaminated rhizosphere soil. **(A)** CAT, **(B)** ACP, **(C)** URE and **(D)** SC. Different small letters indicate significant differences under different Pb concentration treatments and different biochar doses (P<0.05).

**Figure 3** shows the effects of different concentrations of Pb and CSB on soil enzyme activity after 45 days. Compared with that of CK, the catalase (CAT) activity of LB0 and HB0 decreased by 37.63 and 57.31%, respectively (**Figure 3A**). The addition of CSB could alleviate the effect of Pb stress on CAT activity to a certain extent but with a limit; moreover, the recovery effect was best when 5% CSB was added. Similarly, under the two Pb stresses, the addition of 5% CSB had the best improvement effect on ACP activity (**Figure 3B**). The soil ACP activities of LB5 and HB5 were 31.17 U/g and 33.85 U/g, respectively. A continued increase in CSB concentration negatively affected the soil ACP activity. With increasing Pb concentration, the URE activity gradually decreased (**Figure 3C**). After adding CSB, the URE activity (2188.06 U/g) of LB15 was significantly higher than that of LB0, and the URE activity of LB15 was the highest in all treatment groups. Under high Pb concentrations, with increasing CSB concentration, the URE activity slightly increased. In contrast to the activity of the above three enzymes, the soil SC activity increased with increasing Pb concentration, especially in HB0, and the SC activity increased by 34.80% compared with that of the CK (**Figure 3D**). At the same CSB level, except for 15% CSB, the SC activity of the 5000 mg/kg Pb treatment group was higher than that of the 1000 mg/kg Pb treatment group. Taken together, these results showed that Pb stress alone could alter soil enzyme activity and that adding CSB to soil on the basis of Pb stress can improve soil enzyme activity. According to the activity of the different types of enzymes, the best CSB concentration is also not always the same. For CAT and ACP, more than 10% CSB inhibited their activity.

### 3.4 16S rDNA amplicon sequencing data

Bacterial 16S rDNA amplicon sequencing generated 2,355,232 original reads. After filtering low-quality sequences, short-read sequences and chimeric sequences, the clean bacterial reads (1,781,336) were clustered into 11495 bacterial operational taxonomic units (OTUs) at a 97% similarity level (**Table S2**). The dilution curves of the 11 treatment groups ranged from 2344 to 3350, indicating that the 16S rDNA amplicon sequencing data were reasonable (**Figure S1A**). The species uniformity and homogeneity of each treatment were relatively high (**Figure S1B**).

The number of specific OTUs in LB0 and HB0 was lower than that in CK, indicating that Pb stress may inhibit the growth of some bacterial groups in contaminated soils (**Figures S1C, D**). The number of OTUs in the LB2.5 and HB5 groups was higher than that in the LB0 and HB0 groups, respectively, indicating that the CSB concentration promoted the growth of certain bacterial species, and the most suitable concentration for CSB to mitigate Pb contamination varied according to the degree of Pb contamination in the soil.

### 3.5 Variation in rhizosphere bacterial communities

#### 3.5.1 Bacterial community diversity

The bacterial species richness (ACE and Chao1) and species diversity (Shannon and Simpson) were compared (**Table 1**). Compared with those of the CK, the ACE richness index and Chao1 richness index of the low-Pb treatment group were not significantly affected, while the high-Pb treatment significantly reduced the soil bacterial richness (P < 0.05). Compared with those of the low-Pb treatment group, the Shannon diversity index and Simpson diversity index of the HB5, HB10 and HB15 groups significantly decreased (P < 0.05). These results indicate that among all the treatment groups, LB15 had the highest richness and diversity. The results of the above analysis showed that soil community diversity was more sensitive to changes in Pb concentration than to changes in CSB concentration; this was especially true under high Pb conditions, which caused a significant decrease in soil bacterial diversity. The Wilcoxon test analysis shows this result more clearly (**Figure S2**).

**Table 1:** Analysis of Alpha diversity index of soil bacteria between groups.

Treatment	shannon	simpson	chao1	ACE
CK	9.87 ± 0.11a	0.997 ± 0.001a	3553.81 ± 325.04abc	3635.08 ± 341.39abc
LB0	9.71 ± 0.18ab	0.996 ± 0.001a	3642.80 ± 423.22ab	3627.27 ± 396.82abc
LB2.5	9.67 ± 0.14ab	0.996 ± 0.000a	3515.80 ± 338.84abc	3586.01 ± 304.18abc
LB5	9.64 ± 0.46ab	0.994 ± 0.004a	3508.93 ± 317.14abc	3614.55 ± 285.71abc
LB10	9.88 ± 0.18a	0.997 ± 0.001a	3677.98 ± 133.98ab	3795.05 ± 179.31ab
LB15	9.92 ± 0.11a	0.997 ± 0.000a	3917.51 ± 209.94a	3973.20 ± 188.98a
HB0	9.15 ± 0.17abc	0.992 ± 0.002a	3239.23 ± 179.19bcd	3399.14 ± 231.68abc
HB2.5	8.97 ± 0.32bcd	0.991 ± 0.002a	3017.79 ± 286.91bcd	3131.05 ± 291.06cd
HB5	8.24 ± 0.87d	0.976 ± 0.019b	2947.09 ± 412.04cd	3025.69 ± 345.84cd
HB10	8.72 ± 0.47cd	0.985 ± 0.010ab	3114.64 ± 9.37bcd	3198.04 ± 14.01cd
HB15	8.45 ± 0.32cd	0.984 ± 0.005ab	2645.43 ± 193.00d	2757.54 ± 196.67d

Principal coordinate analysis (PCoA) was performed at the OTU level (**Figure S3**). PCoA by weighted UniFrac revealed a significant difference between the high-Pb treatment and the low-Pb treatment. PC1 and PC2 explained 37.92 and 12.31% of the total variance in the soil bacterial community, respectively. When the unweighted UniFrac distance was used, the two axes explained more than 20% of the difference (**Figure S4**).

#### 3.5.2 Bacterial community composition and structure

**Figure 4:**
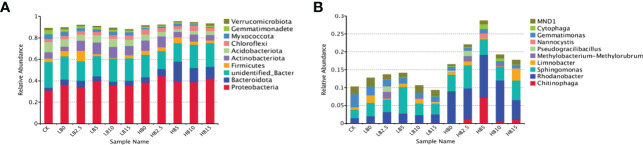
Relative abundance of bacterial community structure in different treatments. **(A)** Phylum level and **(B)** genus level.

The phylum distribution of all the samples showed significant changes in rhizosphere microbial composition in response to Pb and CSB. A total of 83 phyla were detected in the rhizosphere soil samples (**Table S3**), and the top ten were Proteobacteria (37.80%), Actinobacteriota (7.77%), Bacteroidota (7.24%), Acidobacteriota (5.47%), Firmicutes (3.78%), Chloroflexi (3.67%), Myxococcota (2.36%), Gemmatimonadetes (1.76%), Verrucomicrobia (1.23%) and unknown bacterial phyla (20.44%) (**Figure 4A**). Pb stress alone significantly altered the rhizosphere bacterial community structure. Within these communities, the abundance of Acidobacteriota, Myxococcota and Gemmatimonadetes decreased significantly with increasing Pb concentration, while the abundance of Proteobacteria, Bacteroidota and Firmicutes increased in response to Pb pollution. In addition, the abundance of Actinobacteriota and Chloroflexi increased only in response to high concentrations of Pb. Notably, compared with that in LB0, the relative abundance of Actinobacteriota in LB2.5 and LB5 increased by 118.56 and 147.25%, respectively. In addition, compared with that in HB0, the relative abundance of Bacteroidota and Myxococcota in HB5 increased by 280.76 and 138.54%, respectively. The relative abundance of Acidobacteriota and Gemmatimonadetes also increased slightly.

In addition, *Chitinophagales* and *Chitinophagaceae* had higher relative abundances at the order level and family levels, respectively, especially under 5000 mg/kg Pb stress. This indicated that the addition of high concentrations of Pb and 5% CSB could promote an increased abundance of *Chitinophaga*. Moreover, at the family level, the relative abundance of *Sphingomonadaceae* in the LB5 treatment group was 69.46% higher than that in the LB0 treatment group and was the highest among the values in all the treatment groups (**Figure S5**).

To further explore the effects of Pb and CSB addition on rhizosphere bacterial community structure, the community composition of red clover rhizosphere bacteria was analyzed at the genus level. High concentrations of Pb significantly reduced the relative abundance of *Gemmatimonas* and *MND1*. Correspondingly, the relative abundance of *Chitinophaga*, *Rhodanobacter* and *Sphingomonas* was positively correlated with the Pb concentration. After adding biochar, the relative abundance of *Sphingomonas* in LB5 was 95.01% higher than that in LB0 and that the relative abundance of *Chitinophaga*, *Rhodanobacter* and *Nannocystis* in HB5 peaked; in particular, the relative abundance of *Chitinophaga* (belonging to Bacteroidota) was 113.9 times higher than that in HB0 (**Figure 4B**). Notably, the relative abundance of *Chitinophaga* increased in HB5, and the relative abundance of Myxococcota recovered compared with HB0. However, LB5 did not obtain a similar conclusion. In addition, the relative abundance of *Gemmatimonas* and *MND1* was also restored in response to the addition of CSB.

#### 3.5.3 SIMPER analysis

**Figure 5:**
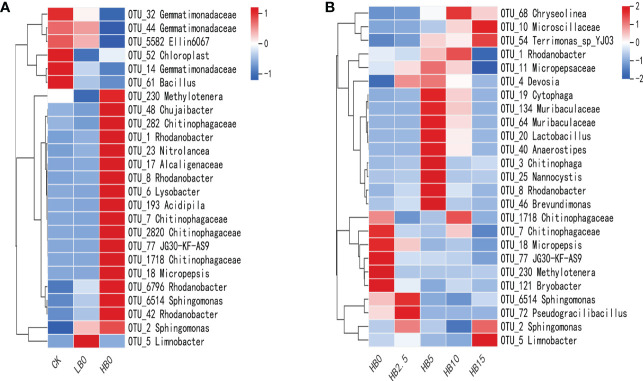
Effects of Pb and CSB on bacterial communities among treatments. **(A)** Most abundant 25 taxa within the treatment identified from CK *vs*. HB0 SIMPER analysis. **(B)** Most abundant 25 taxa within treatments of combined HB0 *vs*. HB5 SIMPER analyses.

SIMPER analysis, combined with hierarchical clustering heatmap displays, was used to show the OTUs that contributed most to Bray−Curtis dissimilarities between treatments. The top 25 OTUs in the CK *vs*. HB0 comparison accounted for 22.02% of the total contribution between treatments (**Table S4**), while the top 25 OTUs in the HB0 *vs*. HB5 comparison accounted for 26.08% (**Table S5**). **Figure 5** shows their contribution to the relative abundance of samples. For the CK and HB0 comparison groups, 25 taxa clustered between samples were divided into three large groups (**Figure 5A**), with top clusters likely to be Pb-sensitive and lower clusters considered to be Pb-resistant or Pb-tolerant. Some of these OTUs identified at the genus level among the resistant Pb toxicity samples were OTU_1 *Rhodanobacter*, OTU_6514 *Sphingomonas*, OTU_6 *Lysobacter*, and OTU_23 *Nitrolancea*.

Proportions of the 25 taxa across treatments that contributed most to Bray−Curtis dissimilarity based on the HB0 *vs*. HB5 SIMPER analysis (**Figure 5B**). According to the rate of CSB, the dendrogram divides co-occurrences of taxa over samples into four main groups. One subcluster at the bottom of the heatmap had five taxa, they were less sensitive to CSB and could survive in a Pb-contaminated niche. In addition, OTU_3 *Chitinophaga*, OTU_8 *Rhodanobacter*, OTU_19 *Cytophaga*, OTU_25 *Nannocystis* and OTU_20 *Lactobacillus* were highly sensitive to 5% CSB treatment. Thus, as visualized in the heatmap, Pb and CSB may exert synergistic effects on the development of the rhizosphere bacterial community, including perhaps an alleviating effect of CSB on Pb toxicity.

Dendrograms illustrate the clustering of taxa among samples.

### 3.6 Regulatory mechanism through which CSB promotes red clover growth and Pb toxicity resistance

**Figure 6:**
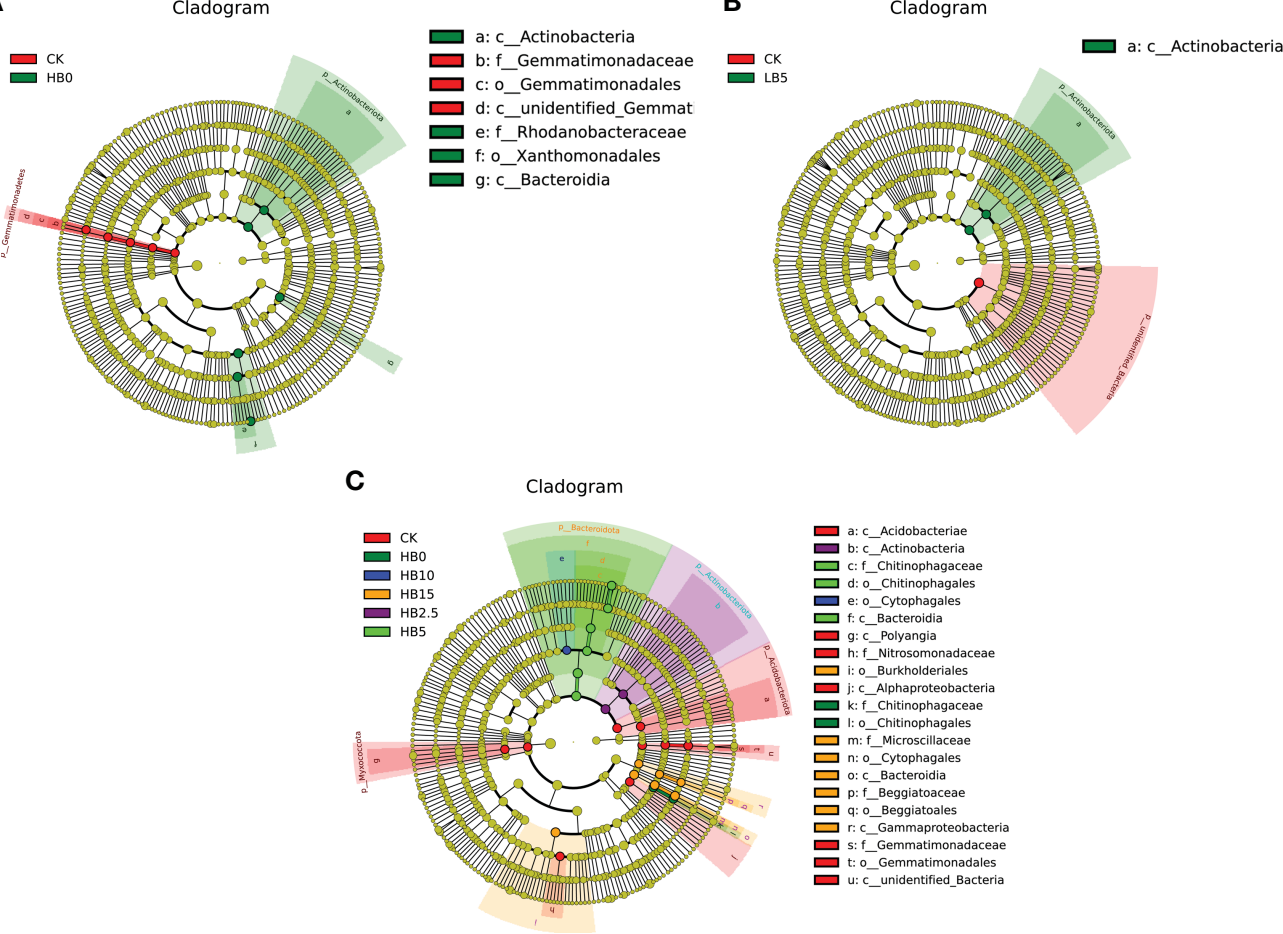
LefSE analysis results of bacterial community members. **(A)** Pb application alone, **(B)** CSB effect under low Pb toxicity and **(C)** CSB effect under high Pb toxicity.

To determine the bacterial biomarkers in the Red clover rhizosphere under the combined action of different levels of Pb pollution alone and Pb pollution together with different CSB concentrations, LDA effect size (LEfSe) analysis (linear discriminant analysis (LDA) score > 4.0, P < 0.05) was used to examine the changes in microbial community structure. In the 11 treatments studied here, after different concentrations of Pb and CSB were added, the abundances of several OTUs were significantly different, especially in the high concentration of Pb pollution and CSB treatment. Under Pb pollution alone, three kinds of bacteria were closely related to high Pb stress: *Actinomycetes* (class), *Flavomonas* (from one order to one family) and *Bacteroides* (class) (**Figure 6A**). Four types of bacteria were closely related to CSB, namely, *Actinomycetes* (one class), *Chitinophaga* (one class to one family), fibrophagocytizing bacteria (one class to one genus) and Proteus (one class to one family) (**Figure 6C**). For low Pb stress, the addition of only 5% CSB resulted in significant differences from CK (**Figure 6B**).

**Figure 7:**
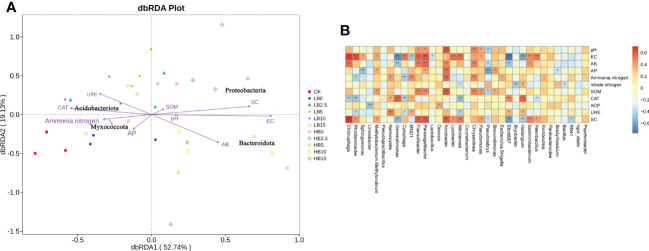
**(A)** Redundancy analysis (RDA) ordination of Pb-contaminated soil bacteria (phylum level) and soil physicochemical properties after CSB amendment. **(B)** Spearman correlation analysis between the soil bacterial community (genus level) and soil physicochemical properties.

Redundancy analysis and Spearman correlation analysis were used to determine correlations between soil physicochemical properties and rhizosphere microbial structure. The RDA results showed that soil physical properties (pH and EC), AK and SC were mainly affected by high Pb levels, and they were more closely related to Proteobacteria and Bacteroidota. Acidobacteriota and Myxococcota are more closely related to ammonium nitrogen, CAT and URE (**Figure 7A**). Plant growth-promoting rhizosphere bacteria identified in this study, such as *Rhodanobacter*, *Sphingomonas*, *Devosia* and *Lysobacter*, were positively correlated with EC, AK content and SOM content. In addition, *MND1*, *Gemmatimonas* and *Nannocystis* were positively correlated with CAT, ACP and URE activity (**Figure 7B**). Notably, CSB was alkaline, and CSB increased the soil pH. The abundance of *Chitinophaga*, *Sphingomonas*, *Devosia* and *Pseudomonas* increased with increasing pH, which led to increases in AK content, nitrate-nitrogen content, SOM content and soil enzyme activity. In addition, **Figure S6** shows the correlations between rhizosphere microorganisms at the phylum level and soil properties.

## 4 Discussion

Pb is classified as a nonessential HM and is highly toxic to plants (Gul et al., 2021). Pb-contaminated soils can affect plants in many complex ways. In most cases, the effects of pollutants on plant growth depend on the concentration of the pollutants. Our previous studies have shown that Pb can inhibit the biosynthesis of photosynthetic pigments in red clover, resulting in a significant decrease in biomass (Meng et al., 2022). High Pb pollution causes a decrease in chlorophyll content and damages chloroplast structure, thereby hindering plant photosynthesis (Šimonová et al., 2007), which is essential for plant growth (Zhang et al., 2022). However, the addition of CSB to contaminated soils alleviated the toxicity of Pb to plant growth (**Figure 1A**). Appropriate doses of biochar have been shown to increase bud growth and root biomass in plants grown in copper tailings (Munir et al., 2021), and similar results have been found in the study of alfalfa (Zhang et al., 2019). This phenomenon may be due to the strong affinity of biochar surface functional groups, which can bind with metals to form metal-ligand complexes and then fix more Pb in the soil (Ho et al., 2017). In addition, the improvement of soil fertility by biochar may be a good explanation for the improvement of plant growth (**Figure 2**). The BCF is an indication of the ability of plants to accumulate HM (Shahbaz et al., 2018). The addition of biochar affects the accumulation and transport of Pb in plants (Huang et al., 2020). Compared with the addition of Pb alone, the addition of CSB reduced the migration of Pb from the soil to plants (**Table S1**). Interestingly, the length of red clover stems was not linearly related to the amount of CSB added. This study showed that adding 5% CSB had the most significant effect on the length of red clover stems, but 10% and 15% CSB inhibited any increase in the length of red clover stems (**Figure 1A**), which means that high concentrations of CSB had a negative impact on crop growth and yield. For example, Liu et al. found that excessive biochar could lead to a decline in crop productivity (Xiaoyu et al., 2013), and similar findings have been obtained in rice (Asai et al., 2009) and maize (Uzoma et al., 2011). Asai et al. found that excessive biochar reduced nitrogen uptake by plants and was related to the fixation of nitrogen by toxic or harmful substances, thereby reducing nutrient uptake (Asai et al., 2009). Many studies have shown that soil nutrient contents are closely related to plant growth (Ahmad et al., 2022; Vaish et al., 2022). Generally, high soil nutrient contents drive plant photosynthesis, stomatal conductance and transpiration, ultimately promoting plant growth (Qi et al., 2021). Biochar is a highly porous carbon structure that is rich in C, N and P nutrients and can be used as a soil amendment (Rahi et al., 2022). Adding CSB can improve the soil pH and soil physical and chemical properties (**Figure 1D**). Biochar can not only desorb nutrients that are present in high abundance and is easily absorbed by plants but also has high nutritional value, which may be the reason why adding raw CSB improves soil physical and chemical properties. (Biederman and Harpole, 2013). Similarly, pot experiments confirmed that an increase in the abundance of several microbes promoted soil fertility after 45 days of incubation in the greenhouse. The increase in available nitrogen, phosphorus and potassium is beneficial to plant growth, especially under HM stress, because the lack of plant-available nutrients hinders plant growth and development.

Soil enzyme activity is closely related to soil nutrients and is a key indicator for maintaining soil fertility-related nutrients (Lin et al., 2021). CAT can detoxify HM, and hydrolases (URE, ACP and SC) are closely related to N, C and P cycling in the soil (Zhu et al., 2022). The activities of URE and CAT in the soil are considered sensitive biomarkers of HM and are widely used to determine the effects of amendments on the mobility and bioavailability of HM in the soil. The increase in CAT activity after biochar addition may be due to the decrease in HM toxicity or bioavailability and may be related to the increase in soil enzyme activity because of the porous structure of biochar particles (Irshad et al., 2022). In addition, studies have shown that the increase in CAT and URE activity is conducive to soil carbon and nitrogen cycling (Li et al., 2021). In our experiment, the CAT activity peaked in LB5 and HB5 (**Figure 3A**), and increasing CSB dose led to decreasing CAT activity, which may be because biochar can absorb a variety of organic and inorganic molecules and may both block the reaction point and inhibit the activity of certain soil enzymes or their substrates (Bailey et al., 2011; Leiyi et al., 2019). The contents of URE and SC were positively correlated with the CSB dose (**Figures 3C, D**), which may be because the sensitivity of CAT was higher than that of hydrolases (Yang et al., 2016). Notably, URE is involved in the soil nitrogen cycle, which involves the hydrolysis of urea into carbonic acid and ammonia, thereby transforming unused nitrogen into a bioavailable form (Liu et al., 2022), which may play an important role in the growth of red clover under high doses of CSB treatment. Sucrose can be hydrolyzed into glucose and fructose by SC, which disrupts glycosidic bonds and affects the transformation of soil organic carbon (Yang et al., 2020). Unlike for the other three enzymes, the application of Pb alone can promote the activity of SC, which may be due to the stimulation of microbial enzyme production by low Pb concentrations or the improvement in the unit respiratory metabolic quotient under HM stress (Belyaeva et al., 2005).

Bacteria are the most abundant and widely distributed microorganisms in the soil; bacteria play an important role in soil formation, material cycling and fertility, and bacterial abundance is restricted by subtle factors in the ecological environment (Jing et al., 2022). Our results were compared with those of Wan et al. under 400 and 1200 mg/kg Pb stress. Wan et al. showed that Pb toxicity had a negative effect on bacterial diversity (Wan et al., 2022). Interestingly, the bacterial diversity under the 400 mg/kg Pb treatment was higher than that under the 1200 mg/kg Pb treatment. This means that lower Pb concentrations can increase bacterial diversity to some extent, but there is a limit. In our study, the effects of CSB on rhizosphere microorganisms depended on the soil Pb concentration and CSB concentration used, and the Pb concentration and CSB concentration determined the degree to which biochar could improve various soil properties. A study by Li et al. showed that high applications of biochar can destroy the environment for microbial growth and thus reduce microbial diversity (Li et al., 2020), which is consistent with our findings in high-Pb soils. However, the high application of biochar in low-Pb soils did not have the same effect. The possible reason is that although a large amount of biochar application destroys the environment for microbial growth to a certain extent, more importantly, it improves soil properties. Under low Pb stress, high-dose biochar had better improvement effects on SOM, AP and ammonia nitrogen, which may create an environment conducive to the growth of more bacteria. Therefore, there was no significant decrease in diversity in low Pb soil. In addition, under the stress of high Pb concentration, the combined action of Pb and biochar may accelerate the activity intensity and metabolic activity of some resistant dominant bacteria, which increases the individual size or quantity difference of this part of dominant bacterial community, reduces the community evenness, and leads to the decrease of diversity.

At the phylum level, Pb toxicity significantly reduced the relative abundance of Acidobacteriota, Myxococcota and Gemmatimonadetes (**Figure 4A**). Studies have shown that acidic environments contribute to stronger phylogenetic aggregation of Acidobacteriota (Conradie and Jacobs, 2021). In addition, acidophilic bacteria have unique physiological characteristics, such as photonutrition, and Pb stress can lead to an increase in soil pH (**Figure 1D**), thus damaging the environment of acidophilic bacteria. Our previous study showed that high concentrations of Pb stress reduce the photosynthetic capacity of red clover (Meng et al., 2022), which may be the cause of the decrease in the relative abundance of acidophilic bacteria under Pb stress. Because of the groups of HM tolerance genes found in Firmicutes and Proteobacteria, these bacteria have been identified as dominant in mining soils. Their ability to live together in contaminated soil was considered to be the reason for the self-healing of HM-contaminated soil in mining areas (Zhao et al., 2019). Actinobacteriota can decompose a variety of organic compounds and can use different carbohydrates as energy sources (Liu et al., 2022). Therefore, the increase in the relative content of Actinobacteriota in Pb-contaminated soil may lead to a large amount of carbohydrate consumption and a reduction in plant available energy reserves, which led to the change in the growth strategy of red clover and the reduction in plant height to maintain life activities. This idea is also supported by a positive correlation between SC activity and Actinobacteriota relative abundance (**Figure S6**). Chloroflexi is an anaerobic bacterium that has been found to play an important role in regulating soil bacterial community composition and forming stable bacterial communities (Kikuchi et al., 2007). Combined with SIMPER analysis, OTU_23 and OTU_77 were found to belong to Chloroflexi, which could stabilize the rhizosphere bacterial community and resist Pb toxicity (**Figure 5A**). In general, low levels of Pb toxicity can create a soil environment conducive to the development of Pb resistance strategies by microbes, while high concentrations of Pb mainly induce physiological adaptations (Renella et al., 2003).

Plant resistance to HM stress depends largely on beneficial interactions between the soil environment and rhizosphere microorganisms. The application of biochar altered the microbial community structure in Pb-containing rhizosphere soils. Along with the effect of 5% CSB on the growth of red clover, the effect of 5% CSB on rhizosphere microorganisms was also studied. *Gemmatimonas* can fix atmospheric nitrogen, increase the availability of nutrients in the soil, improve soil structure, prevent diseases and insect pests, promote plant growth, and increase phosphorus and potassium availability (Liu, 2021). Under the two levels of Pb toxicity, the relative abundance of *Gemmatimonas* decreased but recovered at HB5, which indicates that it may play a role in alleviating Pb toxicity at 5% CSB. Members of the genus *Chitinophaga*, which belongs to the Bacteroidetes, are predominant in soils under high Pb toxicity and have an excellent ability to degrade cellulose and chitin, which can improve the immunity of plants and promote the growth of crops (Kishi et al., 2017). In addition, a study showed that Myxococcota had a predation relationship with *Chitinophaga*. Myxococcota could prey on bacteria through multiple predation mechanisms, which are generally considered to be the key bacteria that regulate the structure and function of the soil microbial community (Dai, 2020). However, HB5 treatment caused a significant increase in the relative abundance of *Chitinophaga* (**Figure 4B**), so the abundance of HB5 Myxococcota recovered, while LB5 did not. This may be the specific detoxification mechanism of CSB under high Pb toxicity. In addition, the potential of *Chitinophaga* yields plant-polysaccharide-degrading enzymes (Funnicelli et al., 2021), which is consistent with the Spearman correlation analysis in this study, indicating that *Chitinophaga* is significantly positively correlated with SC (**Figure 7B**). In summary, Pb toxicity altered the community structure of rhizosphere microorganisms, and the addition of an appropriate amount of CSB could alleviate the effect of Pb and changed the bacterial diversity, which may be due to the addition of biochar to improve the characteristics of lead-containing soil, such as pH, SOM, AP, AK and ammonia nitrogen. In addition, the pore structure and rich functional groups of biochar can also provide more niches for microorganisms. In addition, studies by Zhang showed that biochar may cause soil bulk density to decrease and water retention capacity to increase (Zhang et al., 2020). These changes are conducive to microbial survival and thus change bacterial diversity. Under the combined action of CSB and Pb, the soil microbial community structure composition differed according to the different CSB and Pb concentrations. This may partly explain why the application of appropriate concentrations of CSB can promote the growth of red clover and improve its resistance to Pb toxicity.

## 5 Conclusion

This study showed that Pb at concentrations of 1000 mg/kg and 5000 mg/kg was toxic to red clover, and 5000 mg/kg was more toxic than 1000 mg/kg. Pb toxicity reduced the effective nutrient content of the rhizosphere soil, inhibited the enzyme activity, altered the rhizosphere microbial community structure, and ultimately affected the biomass of red clover. Adding appropriate doses of biochar successfully alleviated the effects of 1000 mg/kg and 5000 mg/kg Pb on the stem length and biomass of red clover, significantly reducing the accumulation of Pb in red clover and promoting increased stem length. In addition, CSB increased the soil pH and EC; increased the soil AP, AK, ammonium-nitrogen, nitrate-nitrogen and SOM contents; and increased the enzyme activity and relative abundance of beneficial rhizosphere microorganisms. CSB (5%) had the best effect on alleviating Pb toxicity in red clover. Under this CSB dose, the addition of biochar increased the pH of the rhizosphere soil; stimulated the relative abundance of *Chitinophaga*, *Sphingomonas*, *Devosia* and *Pseudomonas*; and positively affected the soil available potassium, nitrate-nitrogen and organic matter contents and enzyme activity to maintain plant growth and alleviate Pb toxicity. Future experiments should focus on the molecular response of red clover to Pb and CSB.

## Data availability statement

The data presented in the study are deposited in the online repository, accession number PRJNA907524. Further inquiries can be directed to the corresponding authors.

## Author contributions

LM: investigation, writing – original draft, data curation, and software. YW: data curation and software. MM: software. ZC: software. ZW: investigation. LW: investigation. GC: conceptualization and methodology. XY: conceptualization, methodology, supervision, and funding acquisition. All authors contributed to the article and approved the submitted version.
