# Supplementary figures and images for: Effects of different concentrations of biochar amendments and Pb toxicity on rhizosphere soil characteristics and bacterial community of red clover (Trifolium pretense L.)
Source: Front Plant Sci. 2023 Mar 28;14:1112002. doi: 10.3389/fpls.2023.1112002 (PMC10088434; doi:10.3389/fpls.2023.1112002)

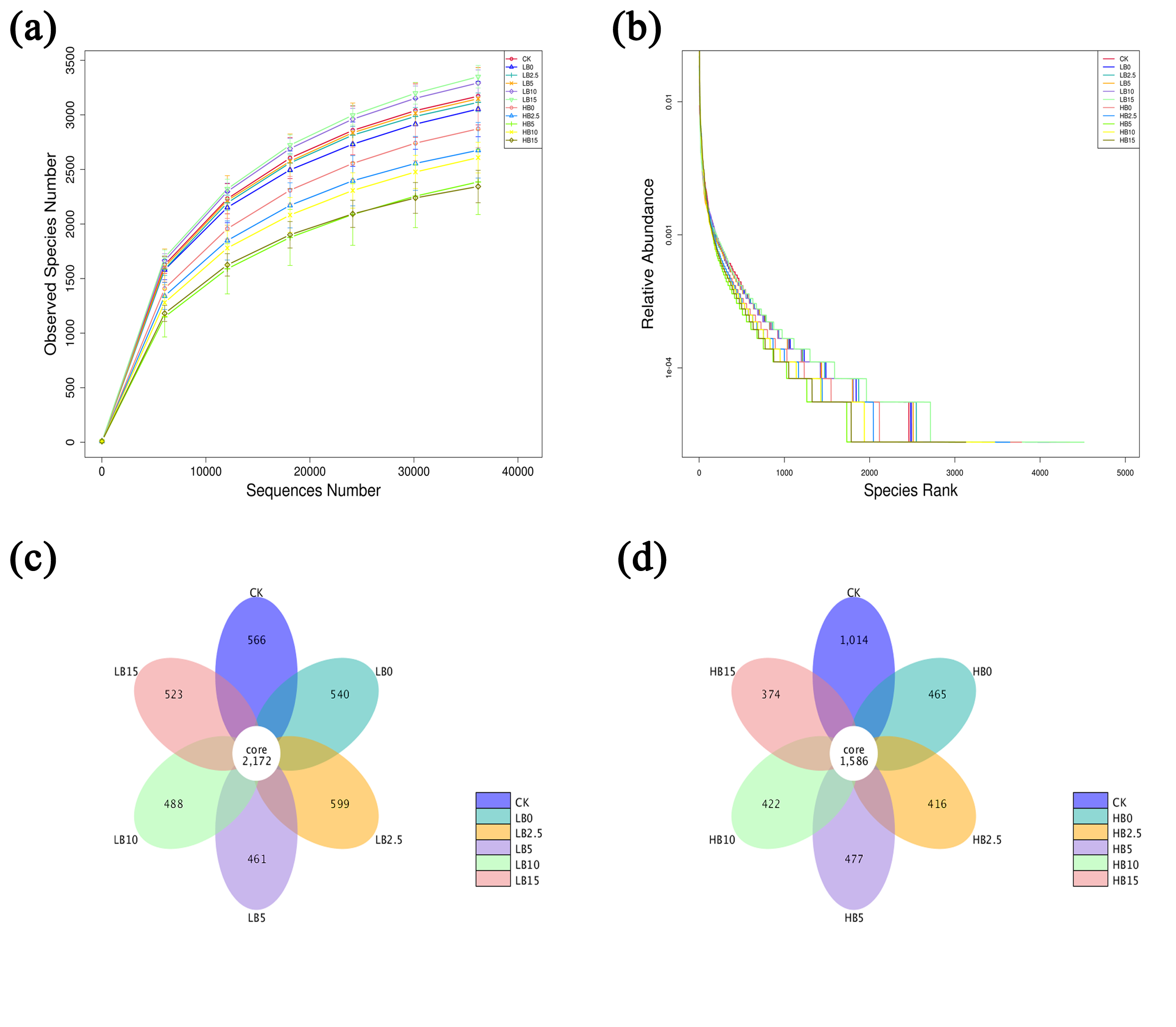

Supplement: Supplementary file 1 [file Image_1.tif]

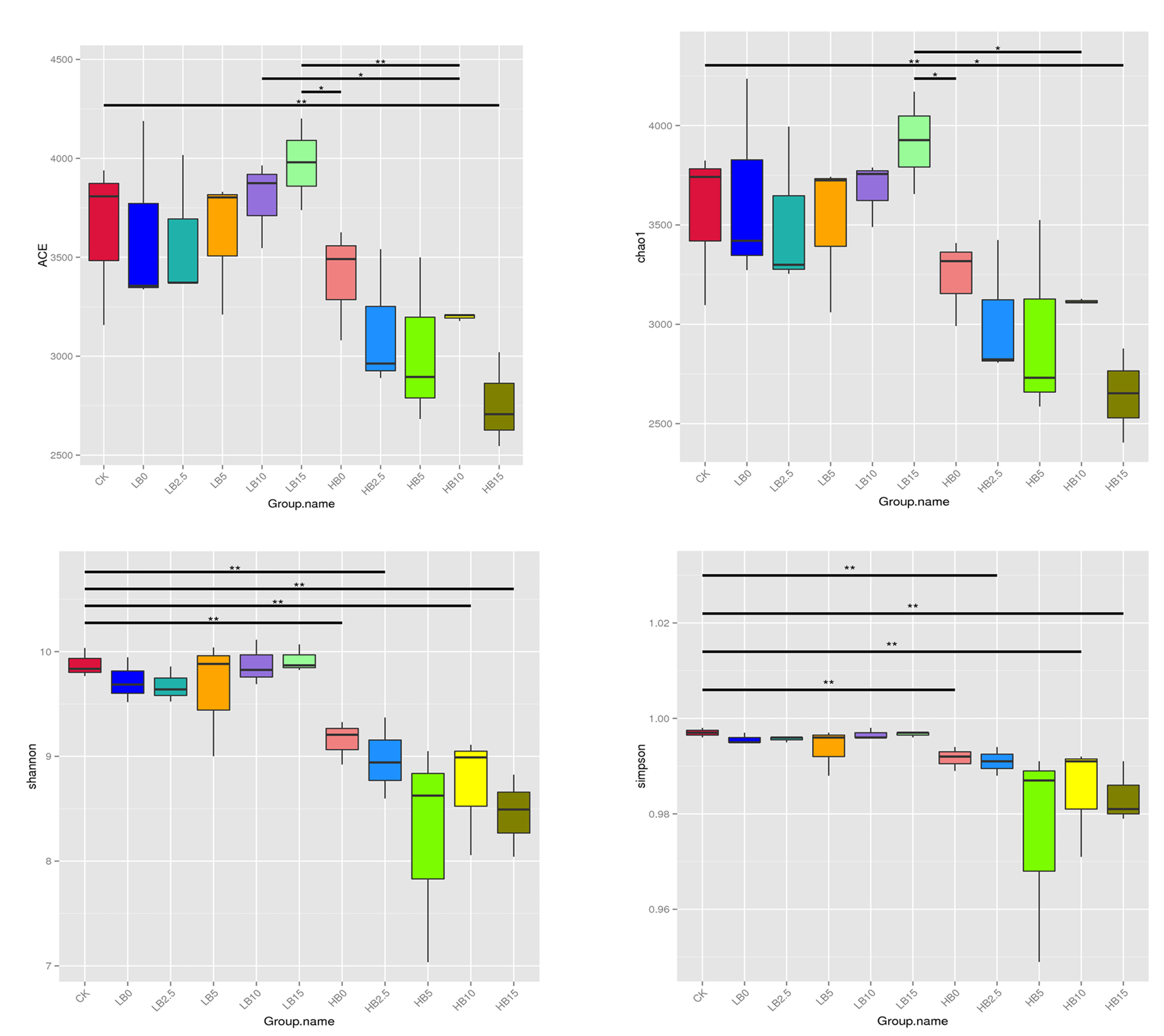

Supplement: Supplementary file 2 [file Image_2.tif]

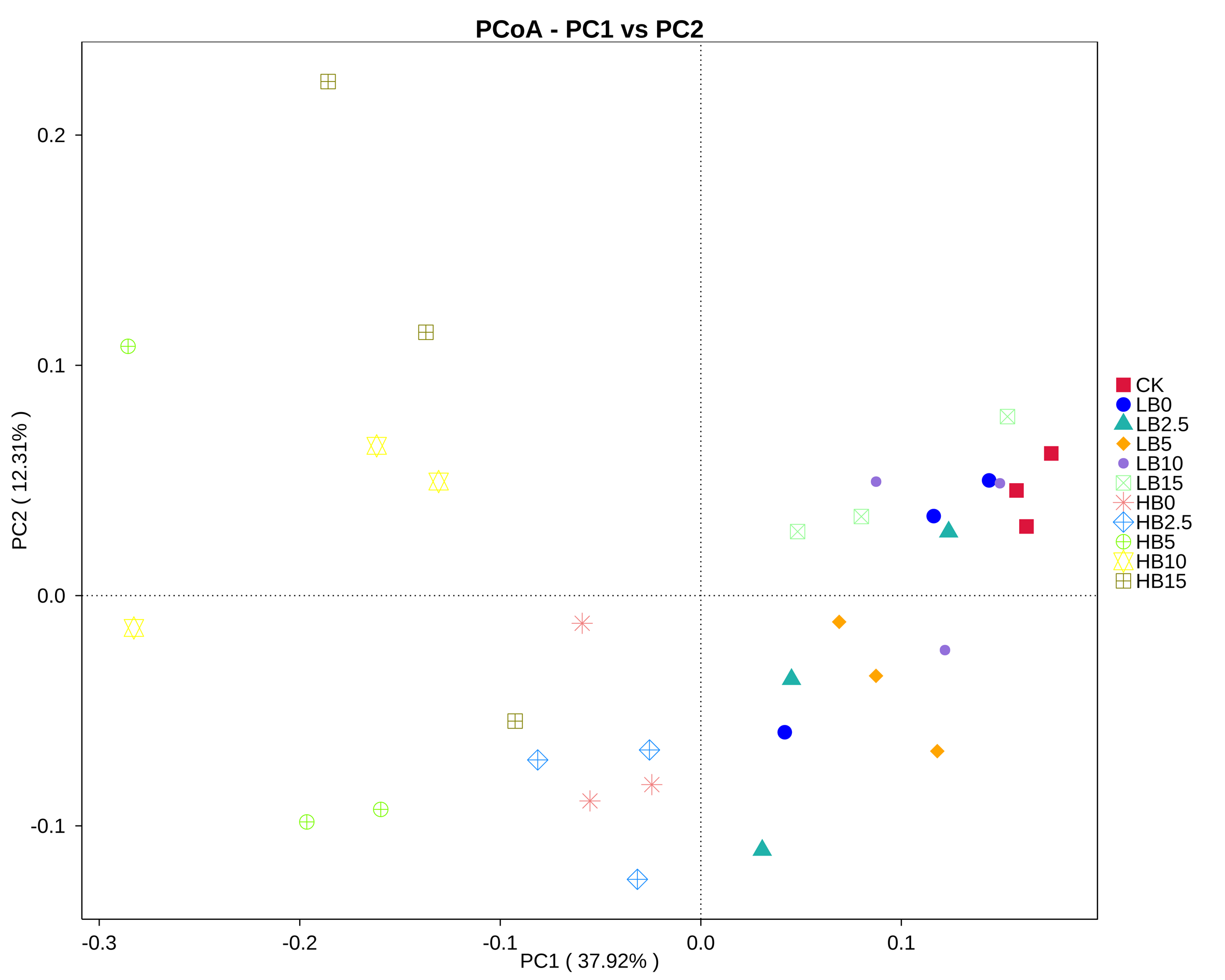

Supplement: Supplementary file 3 [file Image_3.tif]

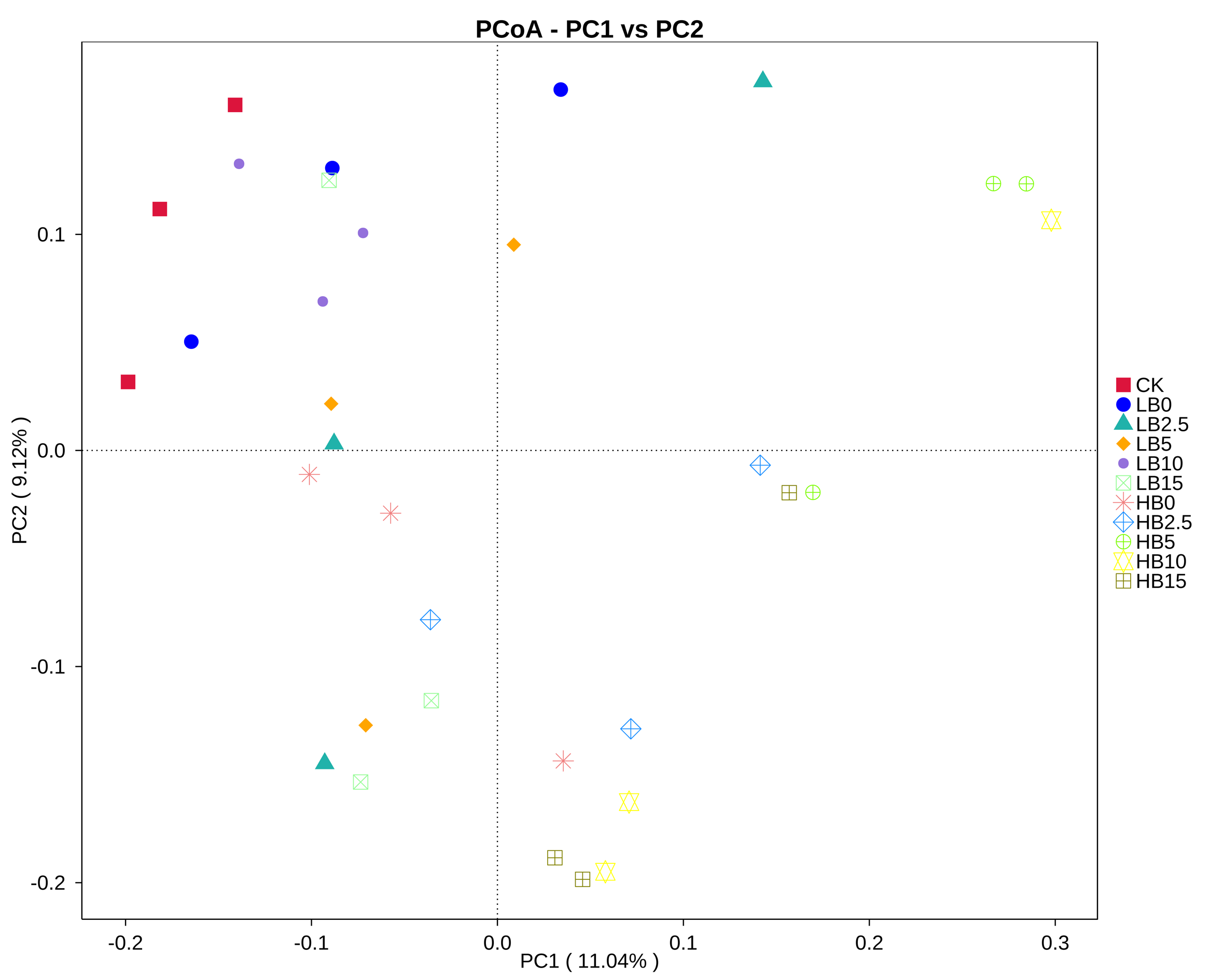

Supplement: Supplementary file 4 [file Image_4.tif]

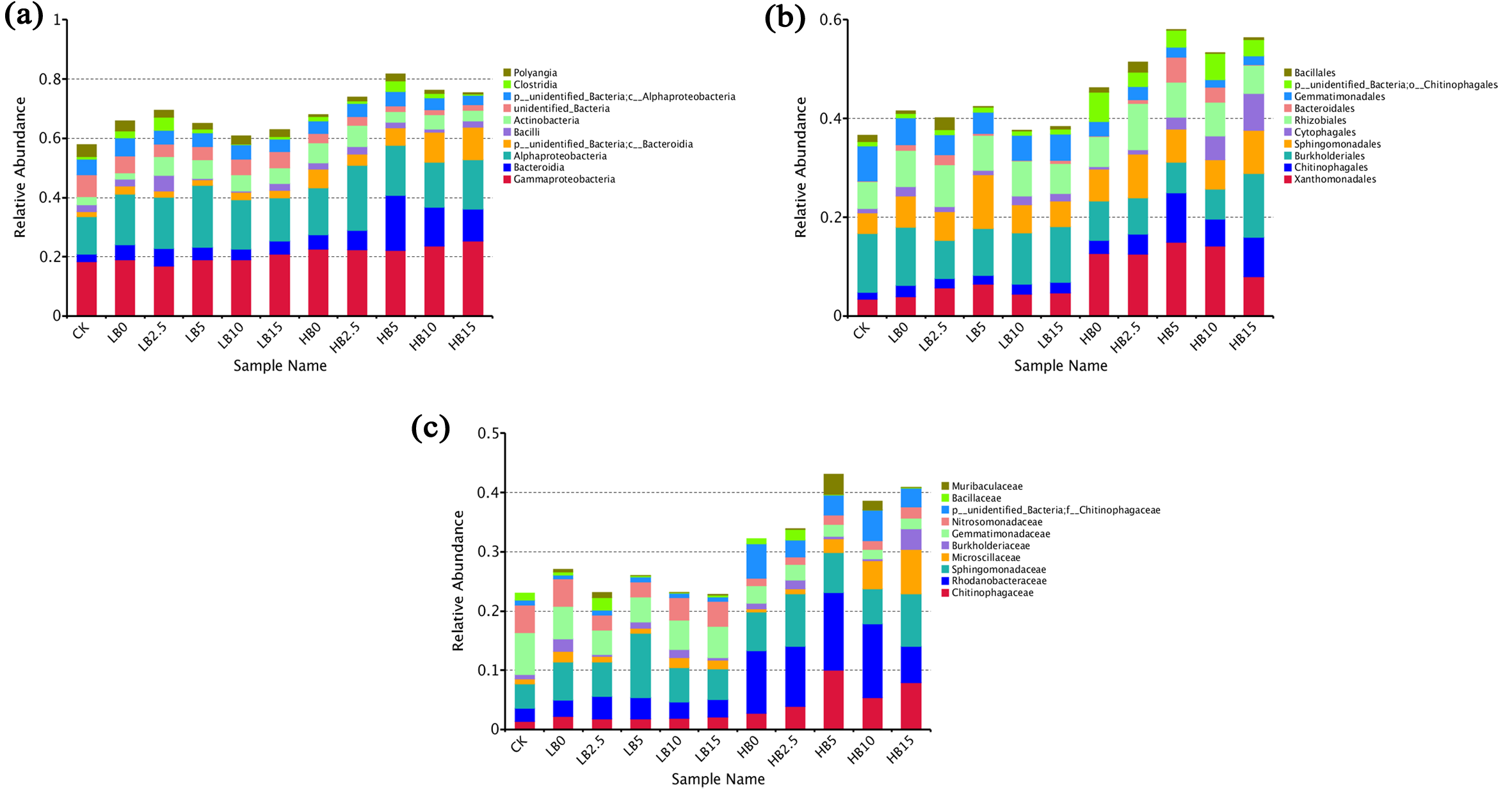

Supplement: Supplementary file 5 [file Image_5.tif]

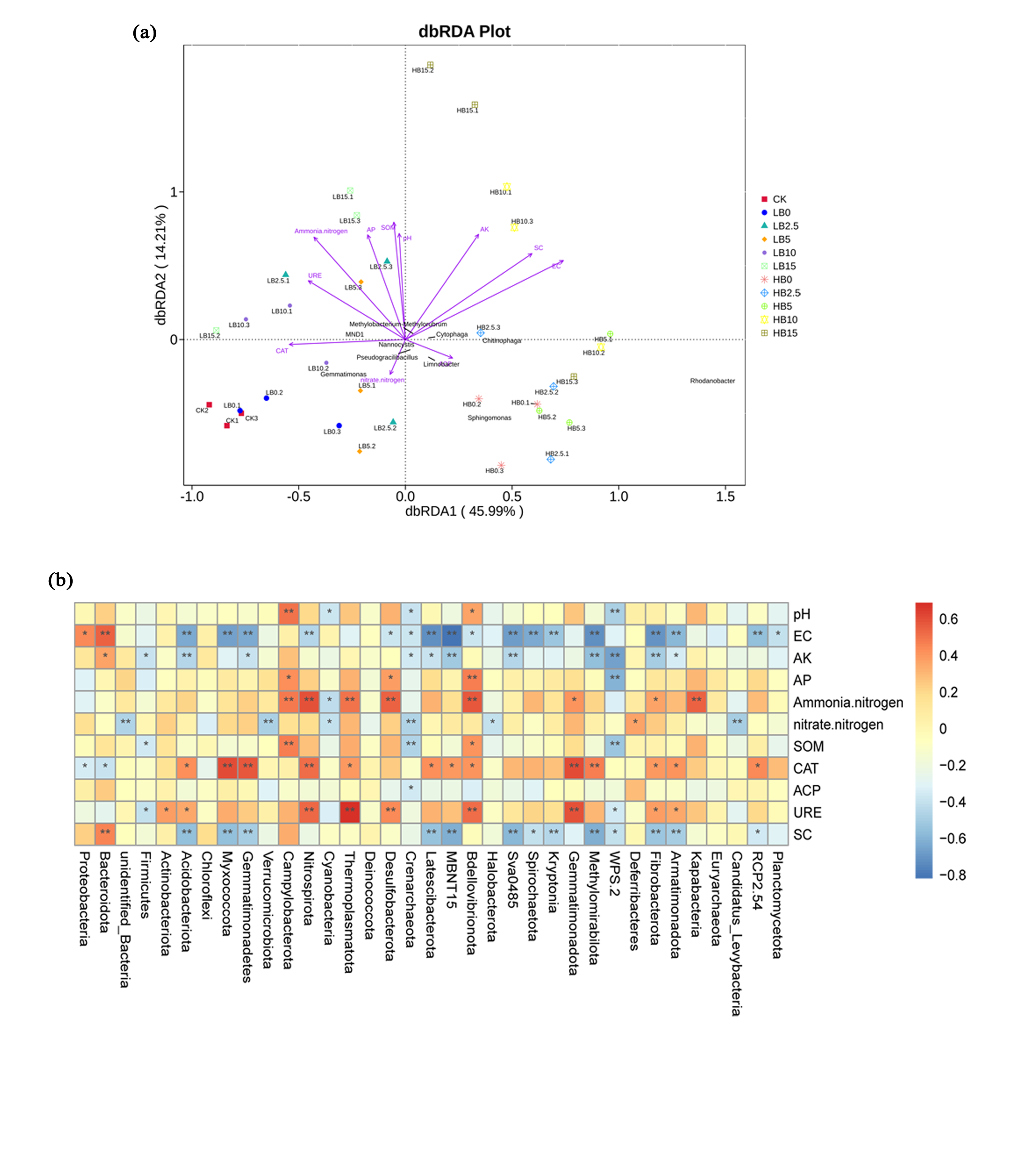

Supplement: Supplementary file 6 [file Image_6.tif]
