# Supplementary material for: Effects of different concentrations of biochar amendments and Pb toxicity on rhizosphere soil characteristics and bacterial community of red clover (Trifolium pretense L.)
Source: Front Plant Sci. 2023 Mar 28;14:1112002. doi: 10.3389/fpls.2023.1112002 (PMC10088434; doi:10.3389/fpls.2023.1112002)
Supplement: Supplementary file 7 [file Table_1.docx]

**Table S1** Pb concentration in soil, uptake by *T. pratense* shoots and roots, translocation factor (TF) and bioconcentration factor (BCF) of plants after 45 days of exposure to Pb and CSB.

| Treatment（mg/kg） | Pb shoot | Pb root | Pb soil | TF | BCF |
| --- | --- | --- | --- | --- | --- |
| CK | 3.70±0.49d | 15.26±2.16d | 63.63±5.64b | 0.24±0.01a | 0.29±0.03a |
| LB0 | 16.60±2.01a | 146.65±3.75a | 687.51±38.40a | 0.11±0.01b | 0.24±0.02ab |
| LB2.5 | 13.55±0.64ab | 112.03±3.13b | 711.31±70.51a | 0.12±0.00b | 0.18±0.02b |
| LB5 | 11.13±0.97bc | 127.78±10.12b | 756.74±99.03a | 0.09±0.00c | 0.19±0.03b |
| LB10 | 7.81±1.23cd | 93.66±5.92c | 774.13±59.85a | 0.08±0.01c | 0. 13±0.02c |
| LB15 | 5.46±0.38d | 149.72±6.12a | 791.28±16.32a | 0.04±0.00d | 0.20±0.01b |
|  |  |  |  |  |  |
| HB0 | 329.70±23.75a | 2011.80±19.39a | 2589.25±76.11b | 0.16±0.01b | 0.91±0.02a |
| HB2.5 | 186.22±11.38b | 2202.93±70.79a | 2621.16±127.95b | 0.09±0.01c | 0.92±0.04a |
| HB5 | 181.94±8.07b | 1949.86±139.30a | 2813.52±41.85b | 0.09±0.01c | 0.76±0.04b |
| HB10 | 144.23±4.40c | 1300.13±151.92b | 3597.22±282.41a | 0.11±0.02c | 0.40±0.04c |
| HB15 | 109.10±6.24c | 1413.15±138.26b | 3369.23±290.15a | 0.08±0.01c | 0.46±0.08cd |

**Table S2** Sequencing data statistics and quality control. Raw PE : original PE reads ; raw Tags : Tags sequence obtained by splicing ; clean Tags : Raw Tags filter low-quality and short-length sequences ; effective Tags : After filtering chimera, the final Tags sequence for subsequent analysis ; Q20 and Q30 : the percentage of bases with base mass values greater than 20 ( sequencing error rate less than 1 % ) and 30 ( sequencing error rate less than 0.1 % ) in Effective Tags ; GC ( % ) : GC base content in Effective Tags ; effective ( % ) : the percentage of effective Tags and Raw PE.

| Sample | Raw PE | Raw Tags | Clean Tags | Effective Tag | Q20 | Q30 | GC% | Effective% |
| --- | --- | --- | --- | --- | --- | --- | --- | --- |
| CK1 | 70,742 | 69,953 | 69,365 | 53,032 | 98.01 | 93.6 | 56.42 | 74.97 |
| CK2 | 65,834 | 64,803 | 64,214 | 47,783 | 98.04 | 93.56 | 56.85 | 72.58 |
| CK3 | 71,249 | 70,438 | 69,859 | 54,852 | 97.99 | 93.54 | 56.25 | 76.99 |
| LB0.1 | 69,278 | 69,232 | 68,896 | 52,248 | 97.87 | 93.27 | 56.19 | 75.42 |
| LB0.2 | 80,763 | 80,380 | 79,896 | 63,292 | 98.03 | 93.61 | 55.91 | 78.37 |
| LB0.3 | 82,747 | 81,482 | 80,770 | 61,317 | 98.6 | 95.21 | 55.24 | 74.1 |
| LB2.5.1 | 58,377 | 57,680 | 57,250 | 43,708 | 97.51 | 92.39 | 55.74 | 74.87 |
| LB2.5.2 | 80,249 | 79,328 | 78,587 | 60,321 | 98.21 | 94.18 | 55.48 | 75.17 |
| LB2.5.3 | 58,200 | 57,576 | 57,050 | 43,291 | 96.63 | 90.54 | 55.94 | 74.38 |
| LB5.1 | 72,675 | 71,668 | 70,878 | 53,473 | 98 | 93.69 | 55.95 | 73.58 |
| LB5.2 | 72,317 | 71,718 | 71,417 | 54,504 | 98.34 | 94.61 | 56.14 | 75.37 |
| LB5.3 | 62,099 | 61,491 | 60,886 | 47,525 | 97.31 | 92.08 | 56.07 | 76.53 |
| LB10.1 | 75,131 | 73,936 | 73,523 | 57,818 | 98.39 | 94.69 | 55.76 | 76.96 |
| LB10.2 | 64,356 | 63,206 | 62,824 | 49,711 | 98.37 | 94.59 | 56.18 | 77.24 |
| LB10.3 | 68,727 | 67,542 | 67,125 | 50,456 | 98.34 | 94.57 | 56.44 | 73.42 |
| LB15.1 | 64,919 | 64,178 | 63,551 | 48,805 | 97.26 | 91.97 | 55.68 | 75.18 |
| LB15.2 | 85,588 | 85,496 | 85,052 | 67,758 | 98.32 | 94.5 | 56.44 | 79.17 |
| LB15.3 | 62,173 | 61,640 | 61,116 | 48,188 | 97.19 | 91.8 | 55.61 | 77.51 |
| HB0.1 | 66,614 | 66,265 | 65,803 | 51,238 | 97.41 | 92.19 | 55.28 | 76.92 |
| HB0.2 | 78,404 | 78,099 | 77,513 | 58,886 | 97.96 | 93.56 | 55.21 | 75.11 |
| HB0.3 | 75,145 | 74,116 | 73,361 | 57,013 | 97.96 | 93.54 | 55.4 | 75.87 |
| HB2.5.1 | 74,718 | 74,106 | 73,706 | 57,203 | 98.32 | 94.53 | 55.46 | 76.56 |
| HB2.5.2 | 69,995 | 69,214 | 68,522 | 52,271 | 97.36 | 92.2 | 54.8 | 74.68 |
| HB2.5.3 | 63,796 | 63,225 | 62,682 | 48,547 | 97.32 | 91.93 | 55.45 | 76.1 |
| HB5.1 | 86,661 | 85,962 | 85,400 | 65,322 | 98.27 | 94.29 | 53.28 | 75.38 |
| HB5.2 | 79,653 | 79,192 | 78,675 | 60,892 | 98.23 | 94.19 | 54.91 | 76.45 |
| HB5.3 | 72,694 | 72,135 | 71,518 | 54,050 | 98.34 | 94.41 | 54.65 | 74.35 |
| HB10.1 | 69,729 | 69,384 | 68,844 | 51,817 | 97.25 | 91.89 | 54.6 | 74.31 |
| HB10.2 | 81,151 | 80,265 | 79,363 | 62,170 | 98.31 | 94.31 | 53.88 | 76.61 |
| HB10.3 | 66,926 | 66,187 | 65,662 | 49,493 | 97.32 | 92.08 | 54.9 | 73.95 |
| HB15.1 | 59,315 | 59,213 | 58,859 | 45,729 | 97.42 | 92.27 | 54.26 | 77.1 |
| HB15.2 | 72,198 | 71,734 | 71,118 | 53,949 | 97.46 | 92.36 | 53.85 | 74.72 |
| HB15.3 | 72,809 | 71,370 | 70,952 | 54,674 | 98.33 | 94.6 | 54.53 | 75.09 |

**Table S3** Statistics of different classification levels of soil bacteria.

| level | quantity |
| --- | --- |
| kingdom | 2 |
| phylum | 83 |
| class | 201 |
| order | 449 |
| family | 594 |
| genus | 1073 |
| species | 561 |

**Table S4** SIMPER analysis of OTU contributions to dissimilarity between CK and HB0.

|  |  | CK | HB0 |  |  |  |  |
| --- | --- | --- | --- | --- | --- | --- | --- |
| OTU | ID | Avg.Abund | Avg.Abund | Avg.Diss | SD | Cum.% | Contrib.% |
| OTU_1 | Rhodanobacter | 0.005197 | 0.062643 | 0.028723 | 0.007524 | 0.053066 | 0.053066 |
| OTU_7 | Chitinophagaceae | 0.001161 | 0.026484 | 0.012661 | 0.003698 | 0.076458 | 0.023392 |
| OTU_14 | Gemmatimonadaceae | 0.018623 | 0.003695 | 0.007464 | 0.005191 | 0.090248 | 0.01379 |
| OTU_2 | Sphingomonas | 0.015039 | 0.028483 | 0.006722 | 0.00189 | 0.102667 | 0.012419 |
| OTU_1718 | Chitinophagaceae | 0.000516 | 0.010127 | 0.004806 | 0.001114 | 0.111546 | 0.008878 |
| OTU_6514 | Sphingomonas | 0.002884 | 0.011979 | 0.004548 | 0.001058 | 0.119947 | 0.008402 |
| OTU_6 | Lysobacter | 0.00305 | 0.011491 | 0.00422 | 0.001198 | 0.127745 | 0.007797 |
| OTU_17 | Alcaligenaceae | 0.000249 | 0.008625 | 0.004188 | 0.000576 | 0.135482 | 0.007738 |
| OTU_61 | Bacillus | 0.008008 | 0.001539 | 0.003747 | 0.002543 | 0.142406 | 0.006923 |
| OTU_42 | Rhodanobacter | 0.002497 | 0.009851 | 0.003677 | 0.001485 | 0.149198 | 0.006793 |
| OTU_52 | Chloroplast | 0.007989 | 0.003437 | 0.003422 | 0.002827 | 0.15552 | 0.006322 |
| OTU_23 | Nitrolancea | 0.000276 | 0.00646 | 0.003092 | 0.00064 | 0.161232 | 0.005712 |
| OTU_77 | JG30-KF-AS9 | 0.000442 | 0.006395 | 0.002976 | 0.00092 | 0.166731 | 0.005499 |
| OTU_44 | Gemmatimonadaceae | 0.010616 | 0.00493 | 0.002843 | 0.001719 | 0.171983 | 0.005252 |
| OTU_48 | Chujaibacter | 0.001567 | 0.007003 | 0.002718 | 0.000566 | 0.177005 | 0.005022 |
| OTU_230 | Methylotenera | 0.003105 | 0.005953 | 0.002659 | 0.002155 | 0.181917 | 0.004912 |
| OTU_2820 | Chitinophagaceae | 0.000516 | 0.005354 | 0.002419 | 0.000979 | 0.186386 | 0.004469 |
| OTU_5 | Limnobacter | 0.006119 | 0.006616 | 0.002377 | 0.002196 | 0.190778 | 0.004392 |
| OTU_32 | Gemmatimonadaceae | 0.005547 | 0.000829 | 0.002359 | 0.001562 | 0.195137 | 0.004358 |
| OTU_5582 | Ellin6067 | 0.006192 | 0.001474 | 0.002359 | 0.000764 | 0.199495 | 0.004358 |
| OTU_8 | Rhodanobacter | 0.000111 | 0.004773 | 0.002331 | 0.000923 | 0.203802 | 0.004307 |
| OTU_6796 | Rhodanobacter | 0.002838 | 0.007427 | 0.002295 | 0.000552 | 0.208041 | 0.004239 |
| OTU_282 | Chitinophagaceae | 0.00082 | 0.005382 | 0.002281 | 0.000397 | 0.212255 | 0.004214 |
| OTU_18 | Micropepsis | 0.003907 | 0.008367 | 0.00223 | 0.001178 | 0.216375 | 0.00412 |
| OTU_193 | Acidipila | 0.000387 | 0.004506 | 0.00206 | 0.00073 | 0.22018 | 0.003805 |

**Table S5** SIMPER analysis of OTU contributions to dissimilarity between HB0 and HB5.

|  |  | HB0 | HB5 |  |  |  |  |
| --- | --- | --- | --- | --- | --- | --- | --- |
| OTU | ID | Avg.Abund | Avg.Abund | Avg.Diss | SD | Cum.% | Contrib.% |
| OTU_3 | Chitinophaga | 0.000147 | 0.066799 | 0.033326 | 0.048064 | 0.068264 | 0.068264 |
| OTU_8 | Rhodanobacter | 0.004773 | 0.0263 | 0.010763 | 0.010313 | 0.090311 | 0.022047 |
| OTU_1 | Rhodanobacter | 0.062643 | 0.074254 | 0.008733 | 0.005519 | 0.108199 | 0.017888 |
| OTU_7 | Chitinophagaceae | 0.026484 | 0.010883 | 0.0078 | 0.003915 | 0.124177 | 0.015978 |
| OTU_19 | Cytophaga | 0.000203 | 0.013325 | 0.006561 | 0.009196 | 0.137617 | 0.01344 |
| OTU_25 | Nannocystis | 0.000166 | 0.013131 | 0.006547 | 0.009707 | 0.151028 | 0.013411 |
| OTU_2 | Sphingomonas | 0.028483 | 0.028401 | 0.004873 | 0.00308 | 0.16101 | 0.009982 |
| OTU_20 | Lactobacillus | 0 | 0.00962 | 0.00481 | 0.003727 | 0.170863 | 0.009853 |
| OTU_10 | Microscillaceae | 0.002046 | 0.011086 | 0.00452 | 0.001592 | 0.180122 | 0.009259 |
| OTU_46 | Brevundimonas | 0.000534 | 0.006828 | 0.003313 | 0.004697 | 0.186908 | 0.006786 |
| OTU_40 | Anaerostipes | 0 | 0.006082 | 0.003041 | 0.002323 | 0.193137 | 0.006229 |
| OTU_230 | Methylotenera | 0.005953 | 9.21E-06 | 0.002975 | 0.002368 | 0.19923 | 0.006094 |
| OTU_18 | Micropepsis | 0.008367 | 0.002497 | 0.002935 | 0.001199 | 0.205242 | 0.006012 |
| OTU_4 | Devosia | 0.01068 | 0.016532 | 0.002926 | 0.001897 | 0.211235 | 0.005993 |
| OTU_134 | Muribaculaceae | 0 | 0.005538 | 0.002769 | 0.00217 | 0.216908 | 0.005672 |
| OTU_64 | Muribaculaceae | 0 | 0.005455 | 0.002728 | 0.002128 | 0.222495 | 0.005587 |
| OTU_6514 | Sphingomonas | 0.011979 | 0.007123 | 0.00244 | 0.001511 | 0.227494 | 0.004999 |
| OTU_77 | G30-KF-AS9 | 0.006395 | 0.001622 | 0.002387 | 0.00105 | 0.232383 | 0.004889 |
| OTU_5 | Limnobacter | 0.006616 | 0.003308 | 0.002173 | 0.002078 | 0.236834 | 0.004452 |
| OTU_68 | Chryseolinea | 0.000313 | 0.004552 | 0.002119 | 0.002127 | 0.241176 | 0.004341 |
| OTU_1718 | Chitinophagaceae | 0.010127 | 0.006073 | 0.002064 | 0.001686 | 0.245404 | 0.004228 |
| OTU_11 | Micropepsaceae | 0.005409 | 0.007547 | 0.002006 | 0.00165 | 0.249512 | 0.004109 |
| OTU_54 | Terrimonas_sp_YJ03 | 0.000461 | 0.00422 | 0.00188 | 0.001624 | 0.253363 | 0.003851 |
| OTU_121 | Bryobacter | 0.004165 | 0.000442 | 0.001861 | 0.001045 | 0.257176 | 0.003813 |
| OTU_72 | Pseudogracilibacillus | 0.003585 | 0 | 0.001792 | 0.002688 | 0.260847 | 0.003671 |
